# Supplementary material for: Seasonality and Vertical Structure of Microbial Communities in Alpine Wetlands
Source: Microorganisms. 2025 Apr 23;13(5):962. doi: 10.3390/microorganisms13050962 (PMC12114076; doi:10.3390/microorganisms13050962)
Supplement: Supplementary file 1 [file microorganisms-13-00962-s001.zip › microorganisms-3520874-supplementary.pdf]

Supplementary material

Seasonality and Vertical Structure of Microbial Communities in Alpine Wetlands

Huiyuan Wang [1,2], Yue Li [1], Xiaoqin Yang [2,3], Bin Niu [2,3], Hongzhe Jiao [2,3], Ya Yang [1], Guoqiang Huang [1], Weiguo Hou [1,4,*] and Gengxin Zhang [2,*]

[1] Institute of Earth Sciences, China University of Geosciences, Beijing 100083, China
[2] Institute of Tibetan Plateau Research, Chinese Academy of Sciences, Beijing 100101, China
[3] University of Chinese Academy of Sciences, Beijing 100049, China
[4] State Key Laboratory of Biogeosciences and Environmental Geology, China University of Geosciences, Beijing 100083, China
*Correspondance: weiguohou@cugb.edu.cn; zhangg@itpcas.ac.cn

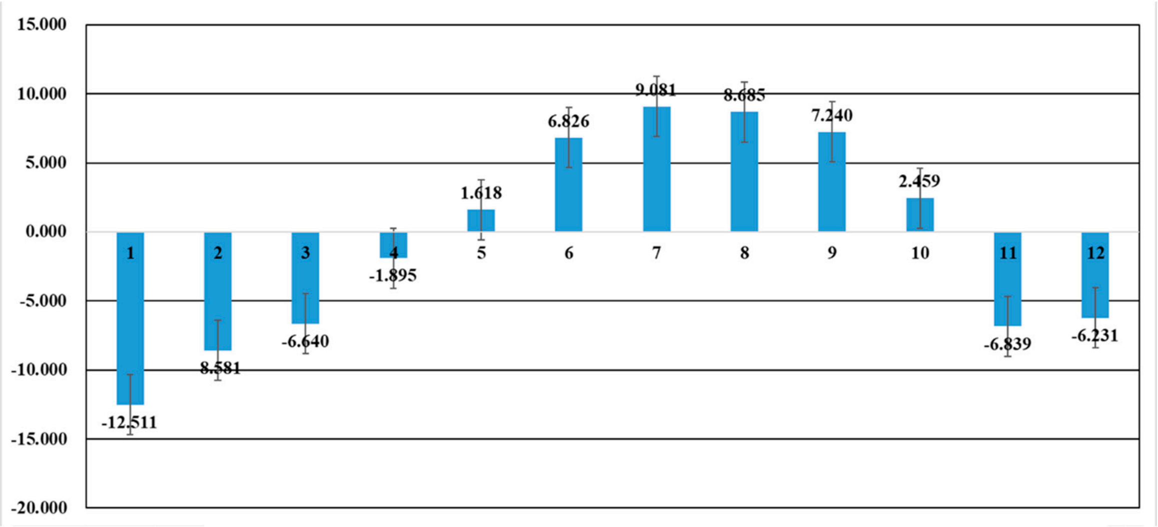

Figure S1 illustrates the monthly average temperature for the year 2017. The X-axis represents the months from January to December, while the Y-axis represents temperature (℃).
